# Volitional Control of Piloerection: Objective Evidence and Its Potential Utility in Neuroscience Research

**DOI:** 10.3389/fnins.2020.00590

**Published:** 2020-06-05

**Authors:** Kenji Katahira, Ai Kawakami, Akitoshi Tomita, Noriko Nagata

**Affiliations:** ^1^School of Science and Technology, Kwansei Gakuin University, Sanda, Japan; ^2^Research Center for Kansei Value Creation, Kwansei Gakuin University, Sanda, Japan; ^3^Graduate School of Letters, Osaka University, Toyonaka, Japan; ^4^Graduate School of Human Sciences, Osaka University, Suita, Japan

**Keywords:** piloerection, goose bumps, volitional control, autonomic, objective measurement, emotion

## Abstract

The volitional control of piloerection has been previously reported in a small subset of individuals. Although this ability may be useful to study the mechanism underlying piloerection, there is little existing research on this ability, neither objective evidence at a group-level, nor information about its stability under experimental constraints. The present study aimed to validate existing findings of voluntarily generated piloerection (VGP) and to examine its potential contribution to neuroscientific research based on objective evidence of this ability. In Study 1, to confirm the characteristics of VGP reported in previous studies and identify individuals with VGP capability, an online survey of VGP candidates was conducted. In Study 2, 18 VGP holders participated in a mail-based piloerection measurement experiment, and the nature of VGP was examined based on the objective data obtained by image-based analysis (GooseLab). Study 1 largely confirmed the characteristics of VGP reported in previous studies, and Study 2 demonstrated VGP at a group-level and provided information about the temporal characteristics of this ability, which supports the utility of VGP in neuroscientific research. For some participants, VGP appeared to be emotionally promoted, which suggests that VGP has some relationship with the emotional nature of involuntary piloerection. Although the studies did not tightly control the environment in which VGP was elicited, the findings nonetheless demonstrate the possible contribution of VGP to elucidating the mechanism of involuntary emotional piloerection and the neural basis of piloerection itself.

## Introduction

Piloerection in humans is an autonomic response observed during a variety of strong emotional experiences, including fear and anger (Darwin, 1872), aesthetic pleasure (Goldstein, 1980), awe (Keltner and Haidt, 2003; Schurtz et al., 2012), and surprise (Maruskin et al., 2012). Dissecting the neural mechanisms of such involuntary piloerection is challenging not only because the researcher must define and differentiate the neural responses, emotions, and stimuli used to evoke the emotions, but also because of the potentially related problem that piloerection in experimental settings reported so far has been low (Benedek and Kaernbach, 2011; Sumpf et al., 2015; Wassiliwizky et al., 2017a, b). For example, piloerection was elicited in a maximum of about 40% of participants in a selection of studies using artistic stimuli such as music (Benedek and Kaernbach, 2011; Sumpf et al., 2015), film scenes (Wassiliwizky et al., 2017a), and poetry (Wassiliwizky et al., 2017b). Therefore, experimental research on physical piloerection requires substantial oversampling in order to achieve an appropriate sample size. To overcome such difficulties, in the present study, we investigated the practical utility of volitional control of piloerection as a means to study piloerection in general.

The voluntary control of piloerection may serve as an efficient method for observing this reaction and contribute to the neuroscientific investigation of piloerection. The autonomic nervous system (ANS) functions largely outside of consciousness, but the abilities possessed by rare individuals or strategies learned through training such as biofeedback methods enable volitional control of ANS activities, including heart rate (Brener and Hothersall, 1966; Bell and Schwartz, 1975; Jones et al., 2015), blood pressure (Lowdon et al., 2011), electrodermal activity (Nagai et al., 2004; O’Connell et al., 2008), and pupillary response (Ekman et al., 2008). Voluntary control of ANS activities is useful in investigating phenomena of interest because it guarantees the generation of robust responses in particular participants and consistently provides opportunities for repeated observations. These abilities or strategies have been used in neuroscience research to examine the centers of volitional ANS control itself (Jones et al., 2015) and specific autonomic activity subject to voluntary control (Nagai et al., 2004).

Previous studies have reported cases of individuals with the ability to volitionally control piloerection (Maxwell, 1902; Lindsley and Sassaman, 1938; Benedek et al., 2010; Uchida et al., 2019). Benedek et al. (2010) objectively recorded piloerection caused at a constant pace, suggesting the potential of voluntary piloerection in neuroscientific research. A recent group study (Heathers et al., 2018) has investigated the overall nature of this ability, characterizing voluntarily generated piloerection (VGP) as produced by volition alone, without any imagination of emotional experiences or stimuli; most strongly formed on the head, neck, or arm; and easily performed by individuals with this ability with a short latency. In addition, VGP was used in activities where involuntary piloerection likely occurred (e.g., music listening, film/TV watching, and book reading), and the execution of VGP was accompanied by some psychological state related to involuntary piloerection (e.g., absorption/immersion, awe/wonder, and detachment). If the group with VGP reported by Heathers et al. could reliably control piloerection, as reported by Benedek et al. (2010), the phenomenon of VGP would have strongly contributed to experimental studies of piloerection for several reasons. First, VGP will assist in elucidating its central control. Modern understanding of the ANS has revealed the orchestrated involvement of cortical structures (Benarroch, 1993). According to this view, studies of pilomotor seizures that are common to temporal lobe epilepsy (Loddenkemper et al., 2004) and direct electrical stimulation of the human brain (Fish et al., 1993) suggest the involvement of cortical areas, particularly in the temporal lobe area, in episodes of piloerection. However, the exact location of the area controlling piloerection remains unidentified (Loddenkemper et al., 2004). The reliable elicitation of piloerection expected through VGP might expand the opportunity for experimental studies to employ less invasive techniques such as fMRI to validate these findings and to reveal the central generator of piloerection. Second, VGP may provide a powerful methodology for laboratory research of emotional piloerection. It reportedly shares situations and psychological effects with involuntary piloerection (Heathers et al., 2018), suggesting that it can be used as a research model to investigate the mechanisms underscoring emotional piloerection.

Considering the potential contribution of VGP to neuroscience research of piloerection, the current study aimed to demonstrate that this ability is repeatedly doable in experimental settings. As existing evidence for this ability at the group level (Heathers et al., 2018) was obtained via self-reports, the physical occurrence of VGP should be objectively verified. It is also necessary to clarify whether VGP can be performed in the presence of various constraints intrinsic to experimental procedures or environments. For these purposes, we replicated the studies of Benedek et al. (2010) and Heathers et al. (2018) in combination. More specifically, we conducted an online survey of VGP-capable individuals to verify the nature of VGP and obtained additional information related to its execution under the constraints of the experimental environment (Study 1). Subsequently, objective and quantitative data on VGP were obtained by a mail-based self-execution experiment to evaluate the feasibility of VGP within the experimental procedures (Study 2).

## Study 1

The purposes of Study 1 were to verify the nature of VGP reported by Heathers et al. (2018), obtain additional information on its suitability for experimental studies, and identify VGP-capable individuals for objective measurements in Study 2. Given the presumed rarity and currently unreliable prevalence estimates of VGP, a large sample was considered necessary. Therefore, large-scale screening and a web survey were conducted by a research company (ASMARQ Co., Ltd). The study was conducted according to the guidelines of the local Ethics Committee of Kwansei Gakuin University. Since the local ethics committee did not request any approval procedure if researchers conformed to committee guidelines, no specific approval procedure was conducted.

### Methods

#### Participants

From an online Japanese panel managed by ASMARQ, 113608 people were randomly invited via e-mail to complete a screening questionnaire titled “Survey about yourself.” Of the 4061 people who responded, 4005 provided complete data. Of these, 112 agreed that they could volitionally invoke piloerection without exposure to cold or emotional stimuli, and were thus identified as VGP candidates (2.8%). Among these candidates, 70 individuals who could participate in the subsequent study were invited, and 61 agreed to complete the survey. In addition, three other VGP candidates identified within the authors’ local community (members of the university community) using a snowball sampling method in advance were asked to participate in the survey. Therefore, Study 1 included a total of 64 participants (32 male and 32 female) aged 17 to 66 years, with a mean age of 42.0 (SD = 11.4) years. Each of them received 500 yen for their participation. Details on the demographic information of the participants are summarized in Table 1.

**TABLE 1:** Demographic information of samples used in Study 1 and Study 2.

	**Study 1: Screening**		**Study 1: Web survey**		**Study 2: Mail-based experiment**		**Population Census data of Japan^a^**
	***n***	**%**	***n***	**%**	***n***	**%**	**%**
**Gender**							
Male	2139	53.4	32 (3)	50	9 (3)	50	49.9
Female	1866	46.6	32	50	9	50	50.1
**Age**							
15–24	86	2.1	3	4.9	1	5.6	12.7
25–34	447	11.2	11 (1)	18.0	3 (1)	16.7	14.4
35–44	922	23.0	22 (2)	36.1	11 (2)	61.1	19.2
45–54	1221	30.5	16	26.2	1	5.6	17.8
55–64	946	23.6	8	13.1	2	11.1	17.2
65–74	383	9.6	1	1.6	0	0.0	18.8
**Total**	4005^b^		64 (3)		18 (3)		

*The information of three pre-specified VGP candidates are enclosed in parentheses. ^*a*^Based on the data from the persons between the ages of 15 to 74 derived from the Population Census of Japan (2015), ^*b*^Respondents who did not provide complete data (*n* = 56) were excluded.*

#### Materials

##### Questionnaire

A questionnaire on volitional piloerection was constructed using an online survey tool (for detailed information, see Supplementary Material). The questionnaire was designed with the intent to largely reproduce the questions regarding the physiological and psychological aspects of this ability in the study by Heathers et al. (2018), with some additional questions (indicated in italics below). The participants were asked about the outline of the ability (*acquisition episodes*, acquisition age, execution methods, and subjective difficulty), bodily reactions (body areas of piloerection and its control, latency and decay, *accompanying physical reactions and sensations*), contexts (activities in which they use this ability, its effects, enhancing involuntary piloerection by this ability, and *situations promoting the ability*), and physical conditions (the influence of *body movement, eye condition, posture*, and breathing). In addition, the participants were presented with a video clip that recorded the procedure for performing volitional piloerection and were asked the extent to which they could evoke piloerection according to the instructions provided in the video.

##### Experimental video

A video clip was created and uploaded online^1^ to present the procedure of the self-performed test for volitional piloerection. Replicating the procedure used by Benedek et al. (2010), both 30 s of rest and piloerection conditions were alternately repeated. The start and end cues for each condition were presented auditorily, and the video included five piloerection trials.

#### Procedure

The 64 VGP candidates who participated in the survey accessed the online questionnaire in their own time and were allowed a single attempt. As part of the survey, they were provided with the detailed information regarding the study purpose. They provided their consent to participate via a digital form and subsequently responded to the questionnaire online.

### Results

#### Identification of the VGP Holders

First, 64 participants were categorized according to the method of generating piloerection, as extracted from the answers about execution methods and acquisition episodes. According to Heathers et al. (2018), VGP is as voluntary as a volitional movement without any imagination of emotional experiences or stimuli. In line with this definition, a subset of participants, including the three pre-specified VGP candidates, mentioned direct volition to elicit piloerection or body-related effort, and they were identified as VGP holders (39% of the participants in Study 1). The other participants (34%) responded to the recall of the antecedents of involuntary piloerection. Two participants (3%) referred to normal involuntary piloerection rather than voluntary piloerection; the remaining participants (23%) did not provide a clear answer. The data obtained from VGP holders are described below and summarized in Table 2.

**TABLE 2:** Number of respondents (percentage) based on the characteristics of VGP.

	**Number of respondents (%)**						
**Outline of VGP**							
Execution method*	Bodily focusing	Recall of involuntary piloerection sensation	Direct volition				
	15 (60)	10 (40)	6 (24)				
Subjective difficulty	Very easy	Easy	Difficult	Very difficult			
	6 (24)	11 (44)	7 (28)	1 (4)			
**Physical response**							
Sites of piloerection*	Arms	Legs	Neck	Back	Face	Chest	Head
	24 (96)	9 (36)	4 (16)	3 (12)	3 (12)	2 (8)	1 (4)
The most intense site	Arms	Face	Legs	Neck	Back		
	20 (80)	2 (8)	1 (4)	1 (4)	1 (4)		
Control of sites and laterality^†^	Sites	Laterality					
	2 (8)	2 (8)					
Latency of piloerection	Less than 5 s	5–10 s	More than 10 s				
	17 (68)	3 (12)	5 (20)				
Decay of piloerection	Less than 5 s	5–10 s	More than 10 s				
	10 (40)	7 (28)	8 (32)				
Associated physical sensations*	Chills	Tingling	Cold sensation	Numb			
	21 (84)	2 (8)	2 (8)	1 (4)			
Associated physical reactions*	Tremor	Muscle tension	Temperature change	Respiratory change	Lacrimation		
	5 (20)	4 (16)	1 (4)	1 (4)	1 (4)		
**Context of VGP**							
Enhancing involuntary piloerection	Yes	No					
	8 (32)	17 (68)					
Activities where VGP is used*	Cold environment	Emotional experience	As a performance				
	4 (16)	2 (8)	1 (4)				
Effects of VGP*	Temperature elevation	Resolving discomfort	Communicating emotion	Enhancing emotion			
	2 (8)	2 (8)	1 (4)	1 (4)			
Promoting situation of VGP*	Cold environment	Emotional experience	Relaxing environment	Fever	Inspiration		
	7 (28)	6 (24)	2 (8)	2 (8)	1 (4)		
**Physical conditions**							
Body movement	Not necessary	Desirable but not necessary	Necessary				
	15 (60)	9 (36)	1 (4)				
Eye conditions^#^	Open-eye	Closed-eye					
	23 (92)	24 (96)					
Posture^#^	Upright	Sitting	Supine				
	23 (92)	24 (96)	22 (88)				
Breathing state^#^	Inspiration	Full lungs	Expiration	Empty lungs			
	19 (76)	21 (84)	16 (64)	18 (72)			

* Multiple answers possible, ^†^Number of respondents answering “controllable,” ^#^Number of respondents answering “practicable.”

#### Characteristics of VGP

Voluntarily generated piloerection holders acquired this ability at 19.1 years old (SD = 8.4) on average. They execute VGP most commonly through “bodily focusing,” followed by “recall of sensation during involuntary piloerection” and “direct volition.” Most of them (68%) regarded this ability more or less easy to execute.

#### Physical Response

Piloerection occurred on the arms in most respondents (96%), followed by legs (36%) and neck (16%). Arms were overwhelmingly reported as the most intense site (80%). As most respondents (80%) reported latencies within 5 s, piloerection occurred quickly, but its decay took longer. Only a limited number of individuals reported that they could control the site and laterality of piloerection (8% for both). The most common physical sensation accompanied by VGP was chills (84%). There were few reports of physical reactions associated with VGP, including tremors (20%) and muscle tension (16%).

#### Context of VGP

Some respondents (32%) reported using VGP to prolong involuntary piloerection, and a limited number of respondents described examples of situations in which they actively used VGP, which included cold conditions (16%) and emotional experiences (8%). Responses on the effects of VGP were also limited, which depicted increases in body temperature (8%) and elimination of physical discomfort (8%). VGP appeared to be easy to execute in certain antecedent situations, including cold environments (28%) and emotional experiences (24%) such as fear, disgust, surprise, and a state of being moved.

#### Physical Conditions

The majority of respondents (60%) reported that they could perform VGP without any physical movement. Eye condition, posture, and breathing did not affect VGP; most respondents mentioned that they could execute this ability regardless of these physical conditions.

#### VGP Execution Test

While only a small number of participants (20%) were able to elicit piloerection in all five trials along the experimental video, the majority of participants (76%) were able to run VGP in more than half of the trials.

### Discussion

The nature of VGP described in this study largely resembled that reported by Heathers et al. (2018). Consistent with Heathers et al. (2018)’s definition, participants reported having the ability to elicit piloerection by their own volition without any imagination or stimuli. During the screening process, 2.8% of the respondents (112/4061) reported voluntary piloerection, and 36% (*n* = 22) of the 61 participants who agreed to take the survey indeed reported their ability in line with the definition of VGP. Considering the incidence of VGP candidates in the screening and the proportion of VGP holders to candidates in the web survey, the prevalence of VGP was approximately 1.0% on a self-reported basis. Most respondents acquired this ability at a young age and executed it easily. VGP was experienced with a short latency of less than 10 s and longer decay, with little control over site or laterality. The arm was the most frequently reported site of piloerection, and most participants experienced the most intense reaction at this site. However, it should be noted that participants may not account for piloerection at all body parts. The participants may have reported major areas in which they frequently noticed piloerection or a strong cutaneous sensation associated with occurrence of piloerection.

Although the context in which VGP was used and its effects were not fully reproduced, the association between VGP and involuntary piloerection was suggested at a different point from that reported previously. In contrast to Heathers et al. (2018) who reported that VGP is performed in activities that cause involuntary piloerection and involves emotional states associated with involuntary piloerection, this study has few such responses and rather emphasizes cold conditions or body temperature response. This result may be due to differences in question format. The previous study (Heathers et al., 2018) used multiple-choice questions, and response items provided the participants with examples of situations where VGP was used and the psychological consequences of VGP. The current study used open-ended questions, which may have increased the load of answering questions and led to fewer answers. Conversely, the present study found that VGP could be boosted by cold and emotional conditions. This result was consistent with the fact that volitional piloerection was easier in situations where involuntary piloerection was likely to be experienced (Maxwell, 1902), suggesting that VGP and involuntary piloerection may share driving factors.

The results regarding the influence of physical conditions (body movement, eye condition, posture, and breathing) support the argument that VGP is suitable for neuroscientific research. Consistent with the study by Heathers et al. (2018), breathing did not seem to affect VGP. A previous study found subtle muscle movements were required to exercise VGP (Benedek et al., 2010), but body movement was not essential for most respondents in this study. Neither open/closed-eye conditions nor posture affected VGP. In general, the participants were not concerned about the influence of physical conditions on VGP, and if this is always the case, VGP would be useful for neuroscience studies that are susceptible to movement artifacts.

However, it should be noted that the results of Study 1 were self-reported and have not been objectively verified. The existence of VGP and its characteristics found in this study need to be confirmed through physical measurements.

## Study 2

Study 2 aimed to confirm VGP ability and further objectively evaluate the practicability of VGP in experimental procedures. For this purpose, we mailed piloerection measuring devices to VGP holders to conduct a self-execution experiment in which the participants measured piloerection on their own. Additionally, Big Five personality traits were measured using the NEO Five-Factor Inventory (Costa and Mccrae, 1992) to test the relationship between VGP ability and personality traits reported in Heathers et al. (2018). As per Study 1, the study was conducted according to the local ethics committee of Kwansei Gakuin University, and no specific approval procedure was conducted.

### Methods

#### Participants

The VGP holders identified in Study 1 participated in Study 2. Due to the number of piloerection measuring devices (15 units) and the limited period of time we were able to reach the participants through the research company, only 15 of the 22 VGP holders identified through screening were recruited. We preferentially selected participants who reported in Study 1 that they easily perform VGP. As the three pre-specified VGP candidates were able to participate in the study at any time, all of three were recruited for Study 2. In total, 18 participants aged 22 to 58 years, with a mean age of 38.4 (SD = 10.2) years, were recruited for the study and received 1,500 yen each for their participation. Details of the demographic information are shown in Table 1.

#### Materials

##### Apparatus

**FIGURE 1:**
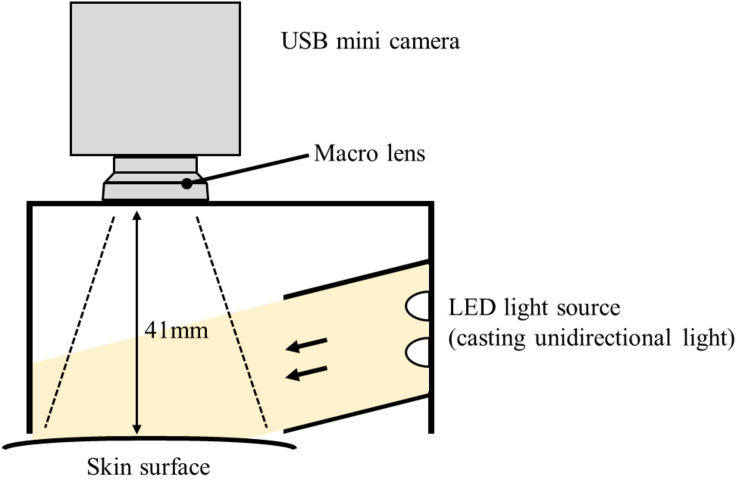
A schematic diagram of the piloerection measuring device.

A measuring device was constructed by the authors to obtain objective data of piloerection. Previous studies have proposed several measuring methods including visual inspection (Craig, 2005), image analysis (Benedek et al., 2010; Uchida et al., 2019), and skin-attached sensor (Kim et al., 2014). In this study, an image-based method proposed in Benedek et al. (2010) was employed since it is easy to introduce, and the analysis program (GooseLab) is provided. This method quantifies the strength of piloerection by image processing of the skin surface. The created device has a hollow plastic structure containing a white LED and a USB mini camera, and the opening on the opposite side of the camera was fixed to the body surface (lower arm) with a belt to permit camera recording of the skin surface (Figure 1).

##### Experimental video

The same video as Study 1 was used to present the experimental procedure.

##### Questionnaire

The Japanese version of the NEO Five-Factor Inventory (Shimonaka et al., 1999) was used to investigate the Big Five personality traits of the participants.

#### Procedure

The measuring device was mailed to the participants along with documents providing a general description of the study, instructions for the experiment, and the questionnaire. The participants who provided written informed consent to participate completed the VGP experiment and questionnaire. Two participants withdrew their consent, and one participant failed to record piloerection data, resulting in 15 complete data sets (12 participants identified by screening and three pre-specified VGP candidates).

In the VGP experiment, the participants accessed the experimental video uploaded onto the web, then performed VGP according to the instructed procedure and answered the extent to which they successfully performed VGP on a 6-point scale: 0 (not at all successful) to 5 (strongly successful). During the experiment, the piloerection measuring device simultaneously recorded the skin surface image and audio of the video. The video included five piloerection trials. Participants performed two sessions (10 trials of VGP).

#### Data Analysis

**FIGURE 2:**
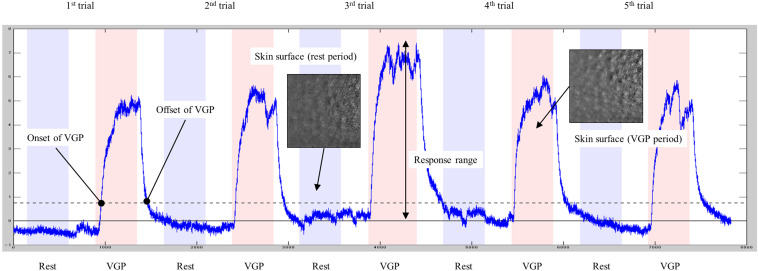
Typical data for the piloerection index. The blue line shows the time course of the piloerection index. The horizontal solid line denotes baseline, and the dashed line represents the threshold (10% of a maximum response range).

In the preprocessing of video data, the analysis area (26.5 × 26.5 mm) was cut out at 15 fps and resized into 288 × 288 pixels. The video data was then analyzed by GooseLab (Version 1.21), and the intensity of piloerection in each frame was obtained. Based on the cues recorded in the audio data, the start and end frames of both rest and piloerection conditions were identified. For each session, a standardized piloerection index was obtained by subtracting the baseline defined as the average amplitude of the five rest periods from the piloerection intensity data (Figure 2).

### Results

#### Subjective and Objective Data for VGP

All 15 participants reported that they were able to generate VGP to a greater or lesser extent in both sessions. The minimum session average (five trials) of subjective ratings for VGP was 0.4, indicating that successful VGP was experienced in at least some trials in every session. The average for all sessions was 1.5. Some participants exhibited little or no piloerection upon visual inspection of video recordings. To identify sessions in which VGP was successfully generated based on objective data, the average amplitude of the piloerection index was calculated for each of the five VGP periods included in each session, and the difference from the baseline (i.e., 0) was tested with a one-sample *t*-test. This resulted in a significant difference in 16 sessions from nine participants (including three pre-specified VGP candidates), and the data from these sessions were further analyzed. To examine VGP latency and decay, time lags of VGP onset and offset were evaluated relative to the time of start and end cues for the VGP periods. According to Benedek et al. (2010), VGP onsets and offsets were identified at the time when the piloerection index exceeded and fell below a threshold of 10% of a response range (i.e., maximum amplitude relative to the baseline in each session). VGP onsets and offsets were detected in all trials included in 16 sessions. However, responses that occurred earlier than the start and end cues for the VGP periods were found for both onsets (10 trials) and offsets (three trials), and these were excluded as premature responses. The onset of VGP occurred at an average of 4.04 s after the start cues for VGP periods (SD = 2.64) and ranged from 0.93 to 15.40 s. The offset occurred at an average of 11.79 s after the end cues (SD = 8.28) and ranged from 1.93 to 55.27 s.

To examine the accuracy of subjectively perceived VGP intensity, we calculated Pearson’s coefficients for the correlation between the subjective ratings and average amplitude of VGP for five trials in each session. As a result, the relationship between the subjective evaluation of VGP and objective data was inconsistent between individuals and sessions. While positive correlations were observed in five participants (*r* = 0.23 to 0.88), they were negative for two participants (*r* = -0.65 to -0.77). One participant demonstrated positive and negative correlations in each of the two sessions (*r* = 0.89 and -0.36). Correlations for the remaining participant could not be obtained because all subjective ratings showed the same value.

#### Personality Characteristics of VGP Holders

The personality traits of VGP holders who elicited measurable piloerection (*n* = 9) were compared with normative data for the Japanese population (*n* = 236) that was obtained from the study of Shimonaka et al. (1999). For each of the Big Five domains, the difference between the average score for these participants and normative data was examined using Welch’s *t*-test. The results revealed a significant difference only in the neuroticism domain, and the score for the participants was higher than the normative data [*t*(8.59) = 3.28, *p* < 0.05, *d* = 1.13]. In the other four domains, the participants scored lower than the normative data (Extraversion, *t*(8.29) = 1.50, *p* = 0.17, *d* = 0.59; Openness to Experience, *t*(8.26) = 0.46, *p* = 0.66, *d* = 0.18; Agreeableness, *t*(8.29) = 1.97, *p* = 0.08, *d* = 0.77; and Conscientiousness, *t*(8.40) = 1.81, *p* = 0.11, *d* = 0.67).

### Discussion

The results of Study 2 objectively demonstrated that the exercise of VGP indeed elicited piloerection in several participants. Other participants who did not show measurable piloerection also reported that they subjectively experienced it. This may be because these individuals had piloerection in body parts other than the forearms. Alternatively, subjective ratings may be formed based on some sort of sensation associated with piloerection, which may have occurred independently of the physical occurrence of piloerection. Several studies that measured both physical piloerection and subjective or related feelings of piloerection (e.g., chills) had reported that subjective and objective data did not always correspond with each other. In terms of co-occurrence, physical piloerection was often absent even though subjective piloerection was reported (Benedek and Kaernbach, 2011; Mcphetres and Shtulman, 2019). Moreover, physiological correlates responded differently for subjective chills and physical piloerection (Wassiliwizky et al., 2017b). Deviation of the subjective experience of piloerection from its actual elicitation may also explain the dissonance between the subjective and objective intensity of piloerection observed in the participants who had measurable piloerection, which emphasizes the importance of objective methods in measurement of piloerection.

In addition, objective data on the temporal characteristics of VGP were provided, demonstrating that this ability was practicable during the experimental procedure. Both quantitatively identified latency and decay were shorter than self-reported measures in Study 1, adding group-level evidence to similar data from the previous study (Benedek et al., 2010). The time course of VGP closely resembled the alternation of the rest and VGP conditions, indicating the feasibility of the experimental procedure in which VGP is repeatedly performed at short intervals.

Regarding the personality traits related to VGP, only the neuroticism of the Big Five personality traits showed an association. This result did not reproduce the importance of openness to experience reported in the previous studies of VGP (Heathers et al., 2018) and chills (McCrae, 2007). Since the mean age and gender composition of the nine participants whose personality traits were examined did not differ significantly from the previous study (Heathers et al., 2018) and normative data (only the information of gender composition was provided), this result is unlikely attributable to a demographic bias of the sample. The theoretical claim which suggests piloerection is a reaction that is inherently associated with fear seems to support the association between neuroticism and VGP (Huron and Margulis, 2010). In addition, the enhanced interoception associated with neuroticism (Deckersbach et al., 2006; Terasawa et al., 2013) may be involved in the acquisition of VGP through subjective awareness of piloerection.

## General Discussion

The present research investigated the feasibility of VGP in neuroscience research with the aim of facilitating the elucidation of mechanisms underlying emotional piloerection. The result demonstrated that VGP is a promising phenomenon to pursue the neural mechanisms of piloerection, mainly through increasing knowledge about physical properties of VGP and confirming voluntary piloerection at the group-level using an objective method.

### Group-Level Evidence of VGP

In this research, individuals with the ability for volitional control of piloerection were identified according to the definition of VGP in a previous study (Heathers et al., 2018), and actual elicitation of piloerection was confirmed by objective measurement with optical recording and image analysis of the skin surface. Since previous research on objectively measured volitional piloerection has reported a single case (Maxwell, 1902; Lindsley and Sassaman, 1938; Benedek et al., 2010; Uchida et al., 2019), this study provides the first population-level evidence of this ability.

The participants recruited through screening (excluding the three pre-specified VGP candidates) revealed the approximate incidence rate of VGP ability. Approximately 2.8% of the screened respondents (112/4061) reported volitional piloerection; from these, about 36% of the participants recruited for Study 1 (22/61) reported their ability in line with the definition of VGP. Among self-reported VGP holders who measured piloerection in Study 2, 50% were able to induce piloerection on their bodies (6/12). Although it should be noted that the population sampled in study 1 did not strictly reflect the demographic characteristics of the Japanese population, these results imply that approximately 0.5% of the population should have VGP capabilities. Since the incidence of VGP is quite low, identifying individuals with this ability may be a major challenge in utilizing volitional piloerection in neuroscience research. The combined questionnaire and measurements employed in this study provide an effective procedure for identifying and verifying VGP holders.

### Feasibility of Utilizing VGP in Neuroscience Research

Voluntarily generated piloerection can be performed in accordance with typical experimental procedures and under the physical constraints inherent in neuroscientific measurements. Participants who voluntarily induced measurable piloerection in Study 2 could demonstrate this ability at a constant pace according to the given cues. They were able to perform this ability repeatedly at short intervals with rapid onset latency. The temporal characteristics of VGP are advantageous for measurement methods typical in neuroscience research, such as block design and signal averaging. Moreover, according to the results obtained in Study 1, most VGP holders would be able to perform this ability irrespective of any physical conditions. These characteristics of VGP are favorable because neuroscientific measurements are susceptible to movement, particularly fMRI, which requires tasks to be performed in a supine position.

### VGP as a Research Model for Involuntary Piloerection

The results on the relationship between VGP and involuntary piloerection highlight the potential contribution of VGP to neuroscience research on involuntary piloerection. VGP has previously been reported to be used in situations where piloerection is experienced involuntarily and produces similar emotional results (Heathers et al., 2018). In this study, self-reported data indicated that VGP could be promoted by some types of emotions (e.g., fear and the state of being moved) associated with involuntary piloerection; this suggested that voluntary and involuntary piloerection share antecedent situations and emotional states. This means that studying the emotional factors that promote VGP may permit the identification of the mechanisms of involuntary emotional piloerection. For example, by independently controlling the execution of VGP and emotional exposure, it may be possible to differentiate emotion-related neural activities that strengthen VGP (and also arouse involuntary piloerection) from those involved in the generation of piloerection itself.

### Limitations

The objective data on voluntarily induced piloerection provided in this study consist of partial evidence obtained by a simple procedure. It should be noted that the environment surrounding the participants and the condition of clothes could not be strictly controlled since the VGP measurements were performed by mail-based self-execution experiments. It was up to the participants to practice the VGP before the experiment as there was no clear instruction regarding practice. This may have potentially affected successful VGP performance. In addition, findings on the physical conditions and the driving factors that enhance VGP are based on self-reports and need to be further verified through experimental studies. Moreover, it will be important to investigate the effects of executing VGP on the activation of other physiological activities. The literature on involuntary piloerection using emotional stimulation suggests that piloerection is accompanied by activity in several ANS substrates (Benedek and Kaernbach, 2011; Sumpf et al., 2015; Wassiliwizky et al., 2017b). Elucidating the physiological activities associated with VGP may allow us to distinguish the activation patterns of the sympathetic nervous system inherent in piloerection from the autonomous activities caused by specific antecedent emotional stimuli.

In conclusion, this study reproduced previous findings regarding the subjective characteristics of VGP and revealed several additional details. Although the large sample used in this study did not accurately reflect the actual demographic structure of the Japanese population, it provided an approximate estimation about VGP prevalence. In addition, objective evidence for the ability of volitional elicitation of piloerection and its temporal characteristics was obtained, supporting the utility of VGP in neuroscience research. VGP will provide reliable observation of piloerection, which contributes to advancing research on piloerection as a physiological phenomenon. Further, our findings on the commonality between VGP and involuntary piloerection, together with findings from previous studies (Maxwell, 1902; Heathers et al., 2018), suggest that this ability may be at least partially utilized in the study of involuntary piloerection. In particular, antecedents common to VGP and involuntary piloerection may give an important role to VGP in the investigation of the mechanism underlying involuntary piloerection. It is hoped that the effects of such antecedents will be experimentally verified in future research.

## Data Availability Statement

The raw data supporting the conclusions of this article will be made available by the authors, without undue reservation, to any qualified researcher.

## Ethics Statement

Ethical review and approval was not required for the study on human participants in accordance with the local legislation and institutional requirements. The patients/participants provided their written informed consent to participate in this study. Written informed consent was obtained from the individual(s) for the publication of any potentially identifiable images or data included in this manuscript.

## Author Contributions

KK and AK: conceptualization and data curation. KK: formal analysis, funding acquisition, visualization, and writing – original draft. KK, AK, and AT: investigation and methodology. KK and NN: project administration and resources. AK, AT, and NN: writing – review and editing.

## Conflict of Interest

The authors declare that the research was conducted in the absence of any commercial or financial relationships that could be construed as a potential conflict of interest.

## Footnotes

1 https://youtu.be/5oQSHG3k2JY
