# Supplementary material for: Volitional Control of Piloerection: Objective Evidence and Its Potential Utility in Neuroscience Research
Source: Front Neurosci. 2020 Jun 5;14:590. doi: 10.3389/fnins.2020.00590 (PMC7290233; doi:10.3389/fnins.2020.00590)
Supplement: Supplementary file 1 [file Data_Sheet_1.PDF]

Supplementary Material

1 Online Questionnaire

1. Please give yourself goose bumps according to the instructions in the video (https://youtu.be/5oQSHG3k2JY). How successfully could you get goose bumps at the pace indicated in the video?

- Completely

- Mostly

- Moderately

- Somewhat

- Hardly

- Not at all

2. How old were you when you acquired or discovered your ability that enables you to give yourself goose bumps voluntarily? Also, please describe the situation in which you acquired or discovered it.

3. Please describe as much as possible about what you are doing in trying to give yourself goose bumps.

4. How difficult is it to give yourself goose bumps?

- Very difficult

- difficult

- easy

- Very easy

5. On which body areas do goose bumps occur when performing this action? Also, please specify the site where goose bumps occur most intensely.

6. Do you have any sensory experiences when you give yourself goose bumps? If the answer is yes, please describe them specifically. Also, please indicate the body areas where the sensory experiences occur and the site at which they occur most intensely.

7. Do you have any other physical reactions when you give yourself goose bumps? If the answer is yes, please describe them specifically.

8. When you start giving yourself goose bumps, how long does it take for the goose bumps to actually occur? Also, when you stop giving yourself goose bumps, how long does it take for the goose bumps to actually turn off?

9. Can you control the location of goose bumps? Also, can you control the side of the body (left or right) where goose bumps occur?

Site

- Yes
- No

Side

- Yes
- No

10. Do you give yourself goose bumps during daily activities? If the answer is yes, please describe them.

11. Are there any reasons to give yourself goose bumps or specific experiences that accompany giving yourself goose bumps? If the answer is yes, please describe them.

12. In the context of involuntary (unintentional) goose bumps, do you make them last longer by your ability to give yourself goose bumps?

- Yes

- No

13. Do you need body motion to give yourself goose bumps?

- No

- No, but it facilitates the goose bumps

- Yes

14. Is it possible for you to give yourself goose bumps with your eyes open or closed?

Eyes open

- Impossible

- Possible

- Very easy

Eyes closed

- Impossible

- Possible

- Very easy

15. Can you give yourself goose bumps in the upright, sitting, or supine position?

Upright

- Impossible
- Possible
- Very easy

Sitting

- Impossible
- Possible
- Very easy

Supine

- Impossible
- Possible
- Very easy

16. Can you give yourself goose bumps under these breathing conditions?

Breathing in

- Impossible
- Possible
- Very easy

Full lungs

- Impossible
- Possible
- Very easy

Breathing out

- Impossible
- Possible
- Very easy

Empty lungs

- Impossible
- Possible
- Very easy

17. Are there any emotional states, situations, or stimuli that prompt your ability to give yourself goose bumps? If the answer is yes, please describe them.
